# Competency-based Education and Training of medical staff. A Programm of the Medical Academy Waldbreitbach: Concept – Implementation – Materials

**DOI:** 10.3205/zma001118

**Published:** 2017-10-16

**Authors:** Eva Hasske, Michael Beil, Katrin Keller

**Affiliations:** 1 Waldbreitbacher Ärzteakademie, Marienhaus Holding GmbH, Waldbreitbach, Germany; 2 Waldbreitbacher Ärzteakademie, Stabsstelle Unternehmens- und Organisationsentwicklung, Marienhaus Holding GmbH, Waldbreitbach, Germany

**Keywords:** competency, competency development, medical training, continuing medical education (CME), speciality training, learning, action competency, professional skills

## Abstract

**Objective: **The aim of the Medical Academy Waldbreitbach is to connect individual and organisational requirements in order to promote an appropriate and multi-locational development of medical competency in the face of the continuously evolving challenges of clinical practice. Integral processes in this are the reduction of organisational learning barriers and the successive integration of competency-oriented learning events in the structures of personnel and organisational development. The modular system for the further development of doctors’ skills serves here as a supplementary and recommendation system for both existing curricula and those defined by regulatory organisations and professional associations.

**Methods: **The Medical Academy’s modular system has a two-dimensional structure. In addition to the axis of biography orientation, the model orients itself around issues relating to the needs of a doctor in any individual professional position, as well as with whom he comes into contact and where his primary challenges lie. In order to achieve better integration in day-to-day routine and a needs-specific orientation of content, the modular system provides a combination of “one, two or three day and two- three- or four-hour training units” depending upon the topic. The transfer of experiential knowledge with the aid of practical exercises is a central element of the didactic model.

**Results: **Through the combined use of summative and formative assessment, the significance of a dialogue-orientated approach in both planning and in the organisational process was highlighted. In feedback discussions and quantitative evaluation sheets, participants identified in particular cross-generational knowledge sharing as a central element for the development of personal values alongside the interdisciplinary transfer of knowledge. The combination of specialist and interdisciplinary topics, for example on team processes or communication, is frequently emphasised, indicating that this had been taught insufficiently and impractically during medical school. Longitudinal evaluations of continuous course units support this, so that the reinforcement of informal learning processes through feedback and exchange of experience is established as an effective and integral learning pattern within the modular system.

**Conclusion: **The of the modular system of the Medical Academy Waldbreitbach – as an institution of the Marienhaus Hospitals Ltd. – is to develop the knowledge, ability and motivation of doctors both individually and professionally. Here, an equally high demand is placed upon the advancement of individual dispositions, attitudes and values, as well as on specialised topics, in order to promote/develop solutions-based and overall medical activity.

## Zusammenfassung

**Zielsetzung: **Ziel der Waldbreitbacher Ärzteakademie ist es, individuelle und organisationale Bedürfnisse zu verzahnen, um eine zeitnahe und standortübergreifende Entwicklung ärztlicher Handlungskompetenz an sich stetig aktualisierende Herausforderungen der Praxis zu fördern. Eine Reduktion organisationaler Lernbarrieren und eine sukzessive Integration kompetenzorientierter Bildungsveranstaltungen in die Strukturen der Personal- und Organisationsentwicklung stellen dabei integrale Prozesse dar. Das Baukastensystem zur weiterführenden Kompetenzentwicklung von Ärzten, dient dabei als Ergänzungs- und Empfehlungssystem zu bestehenden und durch Ärztekammer und Fachverbände definierte Curricula.

**Methodik: **Das Baukastensystem der Waldbreitbacher Ärzteakademie ist zweidimensional aufgebaut. Zusätzlich zur Achse der Lebenslauforientierung orientiert sich das Modell an der Frage, was ein Arzt in der jeweiligen Berufsposition braucht, mit wem er in Kontakt tritt und wo seine primären Handlungsherausforderungen liegen. Für eine bessere Integration in den Arbeitsalltag und bedarfsspezifische Ausrichtung der Inhalte bietet das Baukastensystem eine Kombination aus „1,2,3,-tägigen und 2,3,4-stündigen Trainingseinheiten“ themenabhängig an. Die Weitergabe von Erfahrungswissen unter Hinzuziehung praktischer Übungen ist dabei zentraler Bestandteil des didaktischen Modells.

**Ergebnisse:** Durch den kombinierten Einsatz summativer und formativer Evaluationsprozesse wurde die Bedeutung eines dialogorientierten Vorgehens in der Planung als auch im Veranstaltungsprozess hervorgehoben. In Feedbackgesprächen und quantitativen Evaluationsbögen merken Teilnehmende neben einer fächerübergreifenden, auch insbesondere die generationsübergreifende Wissensweitergabe, als zentrales Element zur Entwicklung einer persönlichen Wertehaltung an. Die Kombination fachlicher mit überfachlichen Themen, bspw. zu Teamprozessen oder Kommunikation, wird häufig mit Verweis, dass diese im Studium nicht ausreichend und zu praxisfern vermittelt wurden, in den Vordergrund gestellt. Längsschnittevaluationen verstetigter Lerneinheiten unterstützen dies, sodass die Stärkung informeller Lernprozesse durch Feedback und Erfahrungsaustausch, als wirksames und integrales Lerndesign im Baukastensystem verankert ist.

**Schlussfolgerung: **Das Baukastensystem der Waldbreitbacher Ärzteakademie – als Einrichtung der Marienhaus Kliniken GmbH – hat zum Ziel, Wissen, Können und Motivation der Ärzte individuell, bedarfs- und praxisorientiert zu entwickeln. Dabei wird der Förderung individueller Dispositionen, Haltungen und Werte ein ebenso hoher Entwicklungsanspruch wie fachlichen Themen unterstellt, um ein lösungsorientiertes und übergreifend authentisch ärztliches Handeln zu fördern/ entwickeln.

## 1. Introduction – Problem Definition

**Figure 1:**
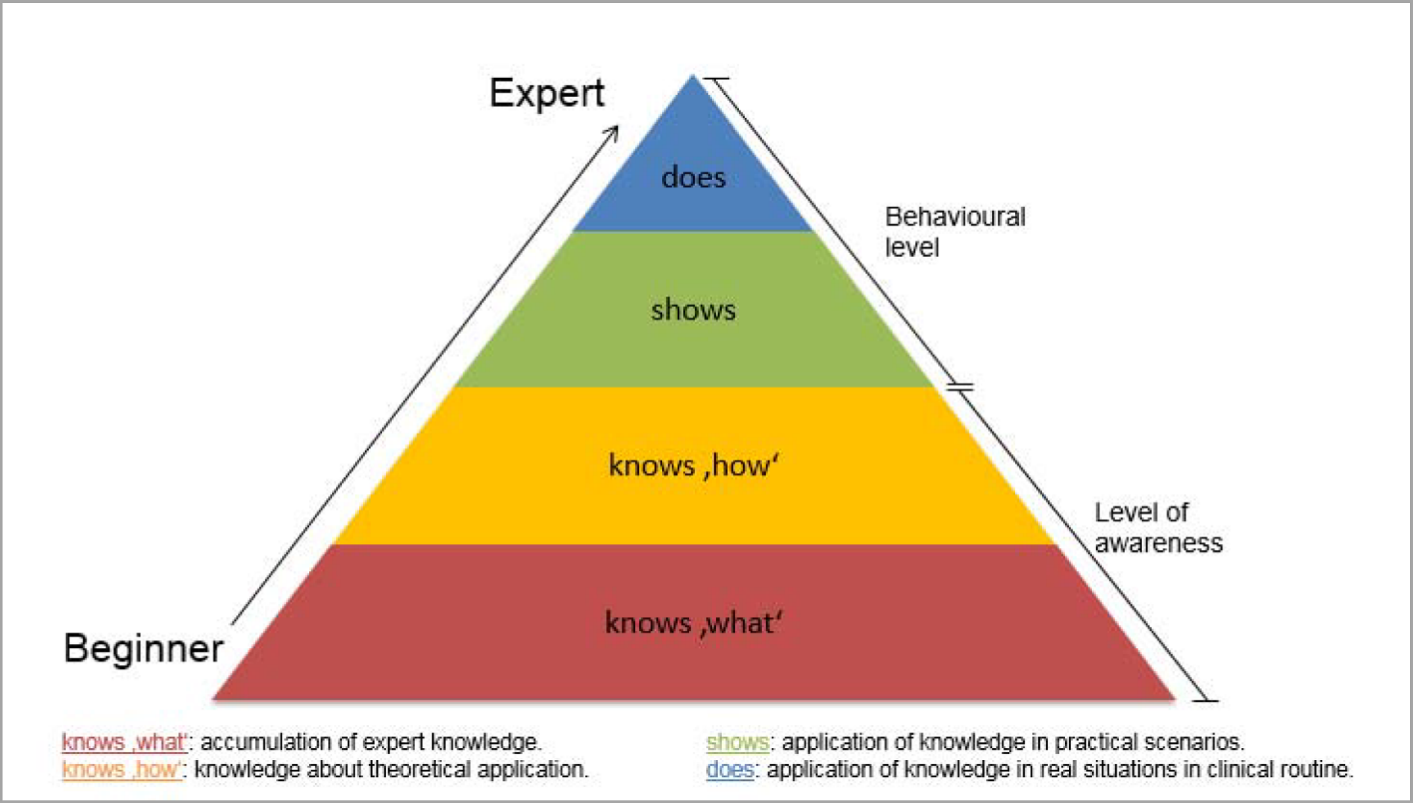
Miller‘s Pyramid for knowledge building and competency development with doctors in the clinical sector (Source: Miller, 1990: http://winbev.pbworks.com/f/Assessment.pdf, last accessed: 18.3.2016)

With regard to the “National Competency-based Learning Objectives Catalogue in Medicine” (NKLM) [http://www.nklm.de] and the revised competency-based items (model) training guidelines (Musterweiterbildungsordnung, MWBO) of the German Medical Association (Bundesärztekammer) [1], [2], practical operational guidelines follow the demand for *larger practical components and an interdisciplinary approach to teaching and knowledge* [3]. The primary objective is the promotion of medical professional activity in the sense of interdisciplinary and overall effective competency [4]. Competencies are accompanied by values, norms and attitudes that are always individual [5]. One’s own and others’ self-reflection processes of individual action provide an essential contribution to the development of the normative and emotional “characteristics” of competencies. Nevertheless, these fields of competency – those of the so-called “soft skills” – frequently remain underdeveloped with doctors, as they are accorded less importance in practice [6]. One possible reason for this can be seen in the variable meaning of qualification and competency (see table 1 (Tab. 1)). Whereas specialised medical knowledge in the context of professional qualifications can be measured and then certified by examinations, a doctor’s practical competency is only acknowledged after his actions have been considered effective by a third party [7]. In order to provide medical (development) potential in an area such as medicine, characterised by the highest levels of innovation, complexity and interdisciplinarity, a change in learning culture is necessary that is focused on the comprehensive development of the individual’s decision-making skills and his capacity to act. A reduction to cognitive structural change should be avoided. On the contrary, the aim should be to “de-formalise” places of learning, to promote practical learning and learning directed at the individual, as well as to orientate learning content on the basis of the outcome (of “*the*” action). Competency-oriented learning in medicine is regarded as an enhancement to the extent that – based upon knowledge and ability – it is able to develop individual potential and values. The Miller Pyramid (see figure 1 (Fig. 1)) shows more simply the components of medical action in clinical practice. In this context, the qualified doctor only achieves competency as a physician with the development of an extensive system of values. 

### 1.1. The Medical Academy Waldbreitbach

The Medical Academy Waldbreitbach (hereafter abbreviated to MAW) was founded in January 2015 with the goal of advancing individual and organisational learning processes. Based on the Christian principles of the Marienhaus group, it is a central service provider of the Marienhaus Hospitals Ltd. The educational task and mandate of the academy are ascribed to the educational sciences. The MAW is counselled and supported by a medical advisory board. Holders of key positions from nursing and other cooperation sectors are invited as required. In order to safeguard the interests of the funding organisation, and for the purposes of quality control, in each quarter discussions on planning and results take place with the chief executives of the Hospitals Ltd.

#### 1.2. Event organisation – Coordination 

All events conducted by the MAW are an integral part of the curriculum within the so-called modular system (for further explanation see section 3). This results in different obligatory events for different medical roles. Obligatory courses were held, for example, which served to promote personal meetings and integration (e.g. “Central Introductory Seminar”/“Emergency Room Training”), or courses which furthered the loyalty and motivation of trainees and doctors in the practical year (e.g. “Trainee Camp”, “Ethics Seminar for Doctors in their Practical Year”). However, the aim is not to establish courses of a general obligatory nature, for which reason offers from senior doctors are communicated as recommendations. The incentive for motivated participation is to be seen more in the time off from work. Thus a natural and subject-oriented “life-long learning” should successively complement the culture of the working process. In order to keep the course costs for the doctors working within the sponsorship low, subsidies from the Clinics Ltd., as well as targeted funding from industry are required. Participation costs for external interested parties differ to some extent. Ordinarily, costs are covered by the individual training budget of a doctor. The participation in events lasting several days is covered by working time. Intensive modules (“learning nuggets”) are frequently attended directly after working hours.

## 2. Project Description – Objectives and Needs of Overall Competency Development

The development of comprehensive competency in medical actions and skills draws upon a broad base consisting of knowledge, ability and motivation, as well as individual values and attitudes already developed during medical training (cf. figure 1 (Fig. 1)) [6]. The necessity of further development of competencies (in a clinical setting) can be explained on three levels:

- educational sciences,
- medical,
- entrepreneurial.

### 2.1. Aims from the perspective of educational science

**Figure 2:**
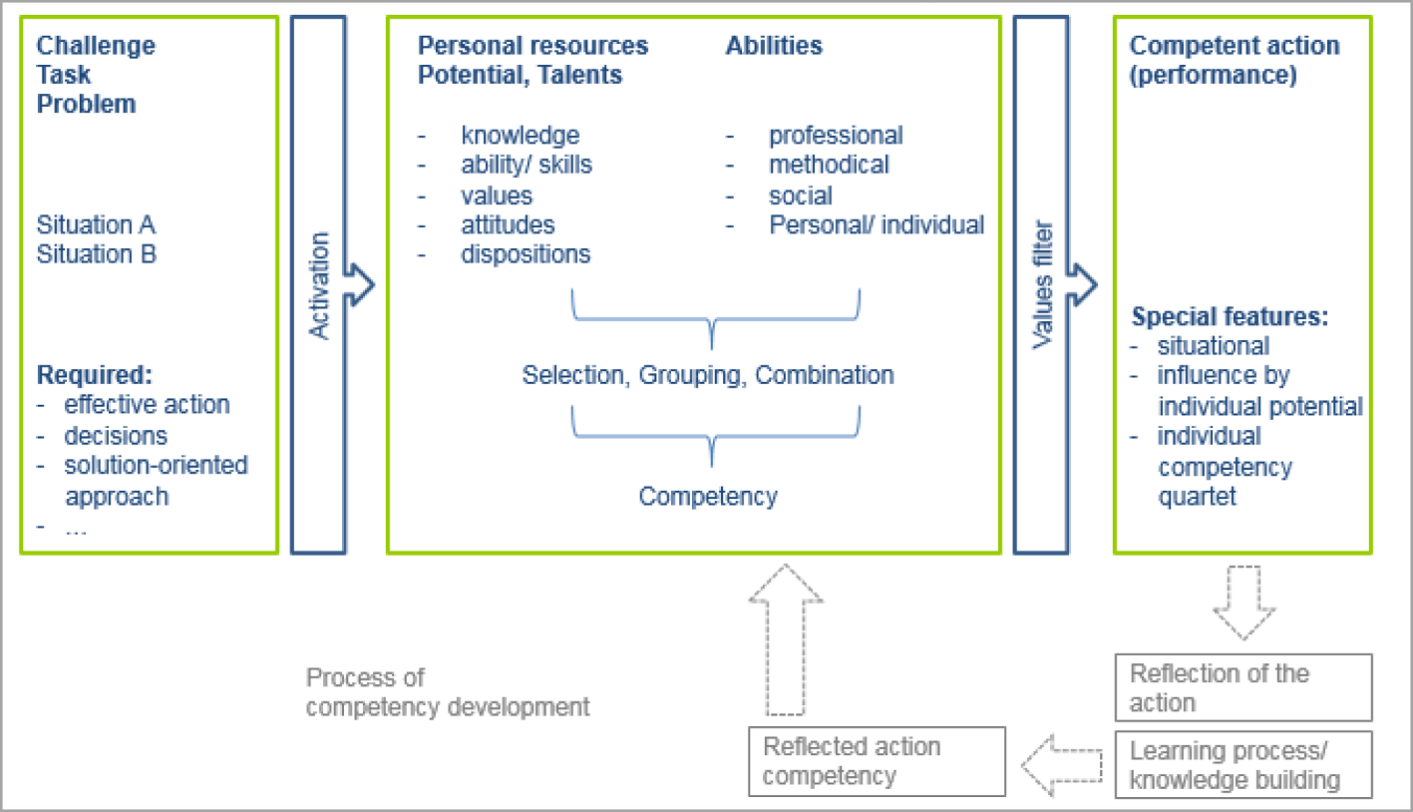
Competency and competency development [following North/Reinhardt/Sieber-Suter 2013 [15]]

Even today, we are confronted with the challenge of breaking through the* ideological reduction of learning to the acquisition of knowledge and expertise, of skills and qualifications* [4]. Specifically, Erpenbeck [5], in agreement with Heyse/Schircks [8], grants medical practice only a limited measure of success in as far as learning processes continue to be declaratively oriented [8]. This means a content-based orientation of learning processes towards the acquisition of factual knowledge without any direct practical reference. Knowledge, in the form of practical action, is only then useful when following the correct organisation and management of declarative and procedural learning processes, whereby the latter describes so-called practical knowledge [9]. The aim of new educational concepts that aim to promote and develop competency must therefore be to combine learning and work processes. Competency development can only be provided if challenges and/or decision situations are acted upon with the help of explicit and implicit knowledge, ability and the required intrinsic motivation as each situation demands [4], [8]. The aim of an interdisciplinary model for competency development must therefore be to reduce structural barriers or break through them as the situation demands. Thus it is necessary to create space for individual self-organised learning processes [10]. Figure 2 (Fig. 2) represents graphically the structure of competency.

#### 2.2. Aims from the medical perspective

Classical methods of education and training in clinical medicine are limited to the transfer of processing abilities to deal with specific problems directly related to patient care. Here, the acquisition of knowledge on physiological and pathological processes, as well as an associated heuristic ability plays a major role [11]. However, it would appear that important areas of medical activity demand additional capabilities in order to adapt to the constantly changing requirements of healthcare. The rising high standard of quality and safety in patient care can only be implemented through an extension of the range of medical training, including interdisciplinary topics – in particular cognitive and communicative competency [12].

For several years, curricula for medical training in various countries with regard to the transfer of skills in various competency areas has been advanced [3], [13]. Using the example of training in internal medicine, table 2 (Tab. 2) shows the areas of competency to be taught in a qualitative comparison of the relevant curricula in Germany (German Medical Association 2015), Great Britain (Royal College of Physicians, London 2012) and the USA (Accreditation Council for Graduate Medical Education – ACGME 2015) in line with the structure of the ACGME. It is apparent that doctors in Germany are allowed hardly any room for development in the area of “audit and independent learning”. Restrictions exist in communicative competency development. Here, the German curriculum limits itself to a sector in which the focus is mainly upon discussions with patients and their families; team processes and interdisciplinary exchange are thus not included to the necessary extent. However, the German Medical Association has set itself the goal of further developing the model training regulations with regard to a broader depiction of areas of competency [14].

#### 2.3. Aims from a business perspective

Medical institutions – whether hospitals, practices, or run by trustees, etc. – have the following in common: it is only the sum of subjective actions that grants a system the capacity for action in the form of ‘competency’. The aim of organisational competency is identical to that of subject-related competency: the increase of reflected action and problem-solving abilities. On a strategic level, therefore, a change of thinking takes place where the areas of study and training successively open up to concepts of organisational development, and thus actively integrate learning into the work process.

#### 2.4. The Issue

Practical experience shows that doctors in training are not sufficiently prepared for many of the challenges of medical practice. In particular, the fear frequently felt during tasks that are not yet (routinely) exercised, or that are completely new (e.g. an operation, a conflict talk, dealing with death, etc.), are the initial considerations of the modular system: what do clinicians require today and tomorrow? The model thus actively tackles the issues of what a doctor requires with regard to his or her professional biography and position and external influences/changes, in order to be able to act authentically and in a goal-oriented manner as each situation demands in everyday professional life. The model sees itself as a complementary recommendation system for existing professional training concepts and further training regulations.

#### 2.5. Objectives of the Medical Academy Waldbreitbach’s modular system

The aim of the competency-oriented modular system is the integral promotion and development of an individual capacity for self-organisation and action. This is based upon the need to support a doctor during the whole of his personal path of learning and education, as well upon the interlinking of external and internal perspectives. Here, competency is understood as the aggregation of all those individual resources that contribute towards self-organised action in any given situation [15], [14].

## 3. Project description – The Medical Academy Waldbreitbach’s Modular System

Increasingly more complex decision-making processes, clinical pictures and situations, a narrowing allocation of staff, as well as uncertainties about the objectives pursued, require the development of comprehensive action competency.

### 3.1. Structure and origin of the model 

The modular system has a two dimensional structure. Alongside curricular distinctions that deal with professional positions (residents to chief physicians) and that stand for the accepted performance level (beginner to expert), there is a division according to areas of competency. Classically, (professional action) competency at a higher level is described as a quartet of specialised, methodical, social and individual competencies. Here, all areas of competency evolve continuously through the reflection process attached to the action process. The awareness for different development requirements of the individual provides the impetus for development and structure of the model. Not least due to the sovereignty of the federal states with regard to content-related form and structure of existing education and specialty training curricula, the aim is to be able to recognise and develop needs both individually and promptly beyond those of specialised qualifications. Challenges in everyday practice, such as one’s own time and organisational management, communication within a team, or the question of premises of ethical medical action in medical law, are referred to here. The model thus attempts to close a deficit between theory and practice by promoting implicit learning within the work process through, for example, experiential learning or exercise scenarios in the field with subsequent (group) reflection. Collegial exchange, which takes place across both generation and position, should thus encourage the development of comprehensive values combined with a strengthening of individual reliability in both decision-making and action. Continuing competency development that manifests itself contextually in everyday issues represents the action-oriented goal dimension of the model.

Contrary to the “common” areas of competency, professional expertise was reinforced by the term “professional competency” in order to signal an active willingness to strengthen existing capabilities. Competency is thus present at an early stage when, for example, a young doctor has already taken blood a few times. Whether, however, the necessary confidence in one’s own abilities/decisions is present, is not necessarily answered.

**Figure 3:**
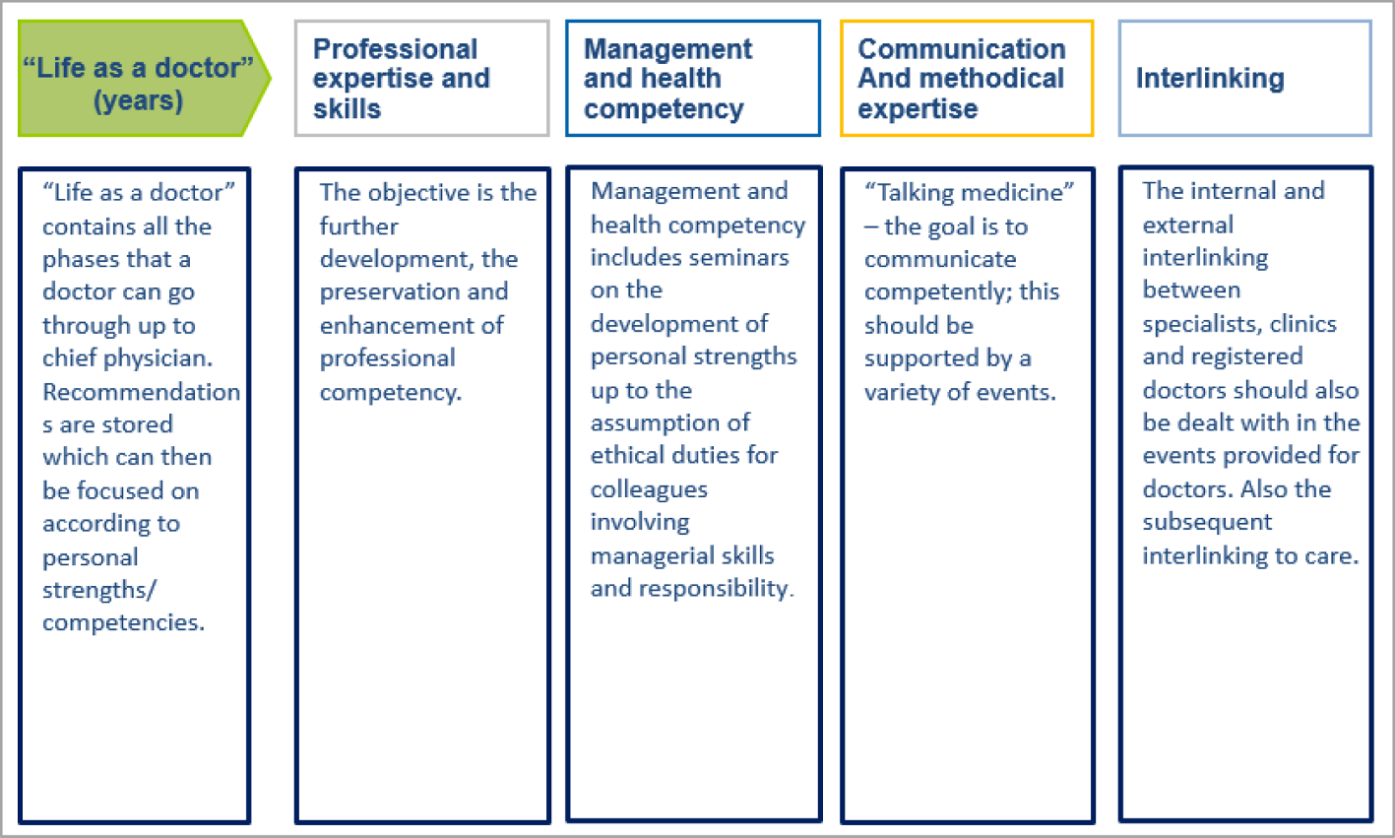
The modular system of the Medical Academy Waldbreitbach – areas of competency

**Figure 4:**
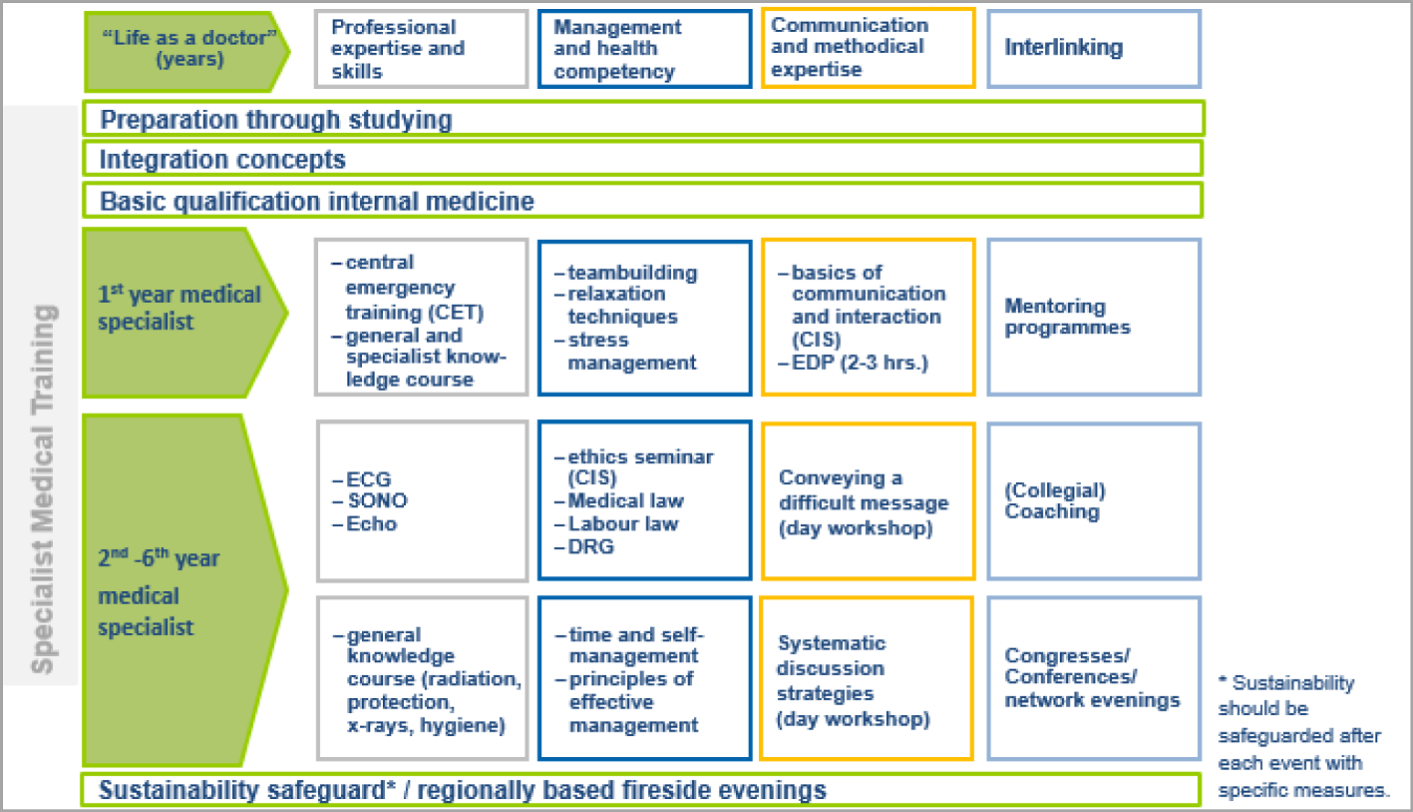
The modular system of the Medical Academy Waldbreitbach – basic structure

At the same time, the field of “managerial and health competency” integrates personal abilities in the sense of a careful perception of an individual’s own physical and mental needs. Here, management competency is not necessarily seen as an hierarchical instrument, but refers rather to relationships between colleagues (including interdisciplinary). The self-awareness that incorporates skills relating to self, time and organisational management is included within this area of competency; this is linked with the understanding and recognition of limitations and dangers. Considerable potential is attributed to learning processes, particularly on a social level. Thus during collegial exchanges – either informally in the work routine or formally organised, for example through fireside evenings or forums, etc. – the sharing of experiential knowledge (e.g. the successes and failures in the treatment of patients with similar courses of disease) takes place. Communicative exchange thus has a positive influence on the individual’s ability and fitness to work through the self-reflective review, break-up and/or change of existing patterns of thought and action. In order to establish a respectful and personalised culture of communication (i.e. not only as far as patients are concerned, but also team colleagues), intra- and interdisciplinary networking plays an essential role alongside the development of communicative and methodical competencies. The fields of competency, and the objectives of the modular system related to them, are graphically presented in figure 3 (Fig. 3) and figure 4 (Fig. 4).

#### 3.2. Target group and content orientation 

As already mentioned, the modular system is geared to the needs of the individual. The learning content of the areas of competency is designed according to years of professional experience; however, no analytical breakdown should dominate. It may be assumed that doctors with longer professional experience have achieved a higher level of competency than, for example, doctors in their first year of specialist training. There are, therefore, separate events for young assistant doctors that are adapted to the respective knowledge of the participants. For those doctors new to the structures of the Marienhaus Hospitals Ltd., there are programmes that serve to familiarise them with corporate culture and structure, and to facilitate their introduction to a new system. Ultimately, current requirements of practice and/or challenges caused by organisational development processes provide the basis for learning contents. These are identified by the doctors themselves and/or those responsible within the company. Effective communication and cooperation between personnel managers (e.g. executive medical staff, training personnel, medical directors, department of human resources) and the overall educational institutions (e.g. philosophical-theological college in Vallendar [majority shareholder: Marienhaus business group/private university], Medical Academy Waldbreitbach) thus becomes a prerequisite of needs-oriented educational opportunities. Further to this, the following factors come in for special consideration: learning process (objective), approach to learning (implementation of methods/didactics) and regulation of learning (when, where, how long). Whether or not a learning unit is obligatory for a selected target group depends upon the topic, or is oriented towards the wishes of the sponsors, as well as the socio-political situation.

#### 3.3. Implementation of the model

**Figure 5:**
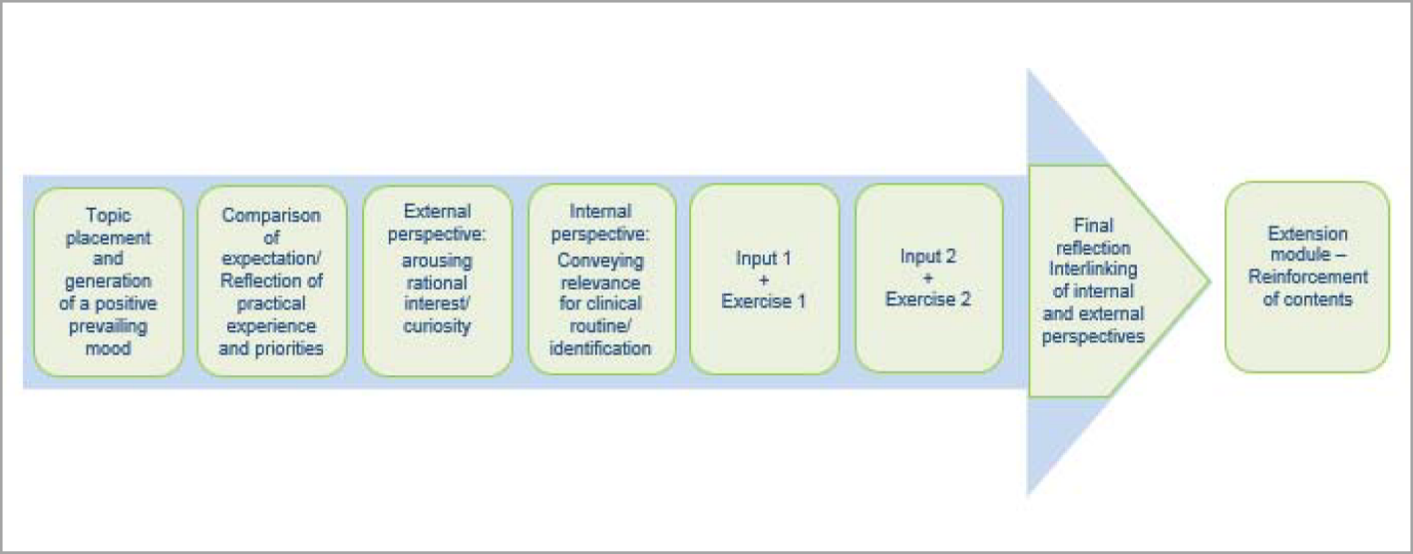
The modular system of the Medical Academy Waldbreitbach – process of modular events

The introduction of the model during the initial phase of the Medical Academy Waldbreitbach at the beginning of 2015 signalled the practical implementation of the modular system. As a result, a combination of “one, two or three day and two- three- or four-hour training units” (depending upon the topic) provides structure. Considering that these are primarily organised within the institutions, an efficient and motivation-increasing integration directly into everyday work routine is possible. The didactic approach of various forms of learning further encourages the desired learning outcome: utilising theoretically acquired knowledge reflectively, situationally and solution-oriented as an “ability” [cf. figure 2 (Fig. 2)] in practical application. As well as a restriction of formal learning arrangement (organised learning in educational institutions), space is created for non-formal learning (incidental learning, inside and outside of educational institutions), for example through expert groups, mentoring programmes, joint “business lunches” or “talks over coffee”. The sharing and exchange of practical experiences are considered essential so that instructors (the practitioners of subjects being taught, e.g. specialists, consultants staff positions, …) are required to proceed in a consultative, moderating and cooperative way (for example joint reflection, case reviews, etc.). Feedback and an open culture of communication represent integral components of the didactic approach and are guaranteed time slots that are scheduled into the event. The next consideration of the modular system is, therefore, to provide an event designed especially for instructors in the form of a “didactic methodology workshop”. Elevating the instructors’ activities to a “quality feature”, and offering a series of training units alongside a more or less official application procedure, are emphasised in order to increase the motivational components that are certified and that go beyond the current feedback talks with those responsible within the medical academy. The modular structure is shown in figure 5 (Fig. 5).

##### 3.3.1. Knowledge building

The plurality of forms of learning and learning styles (visual, auditory, communicative, kinaesthetic) pose structural challenges to the selected setting. Efficient learning that fits the needs of all participants is unlikely in a classic seminar situation with an homogenous learning process [4]. For the methodical-didactical approach of the module, a pool of methods is necessary (exchanges of experience, quizzes, the use of devices and media, storytelling, group units, case reviews, …) that that suits the needs of all learning types. In this context, a change of the requirements and demands that instructors and training personnel (have to) prepare themselves for is needed. Their professionality is characterised by situational competency that aims at a self-directed learning process for the learner.* The learner/trainee becomes the “learning companion”*, he provides situational and methodical support, e.g. via expectations and demands, motion sequences, the use of materials, etc., and moderates the group [10].

##### 3.3.2. Qualification

**Figure 6:**
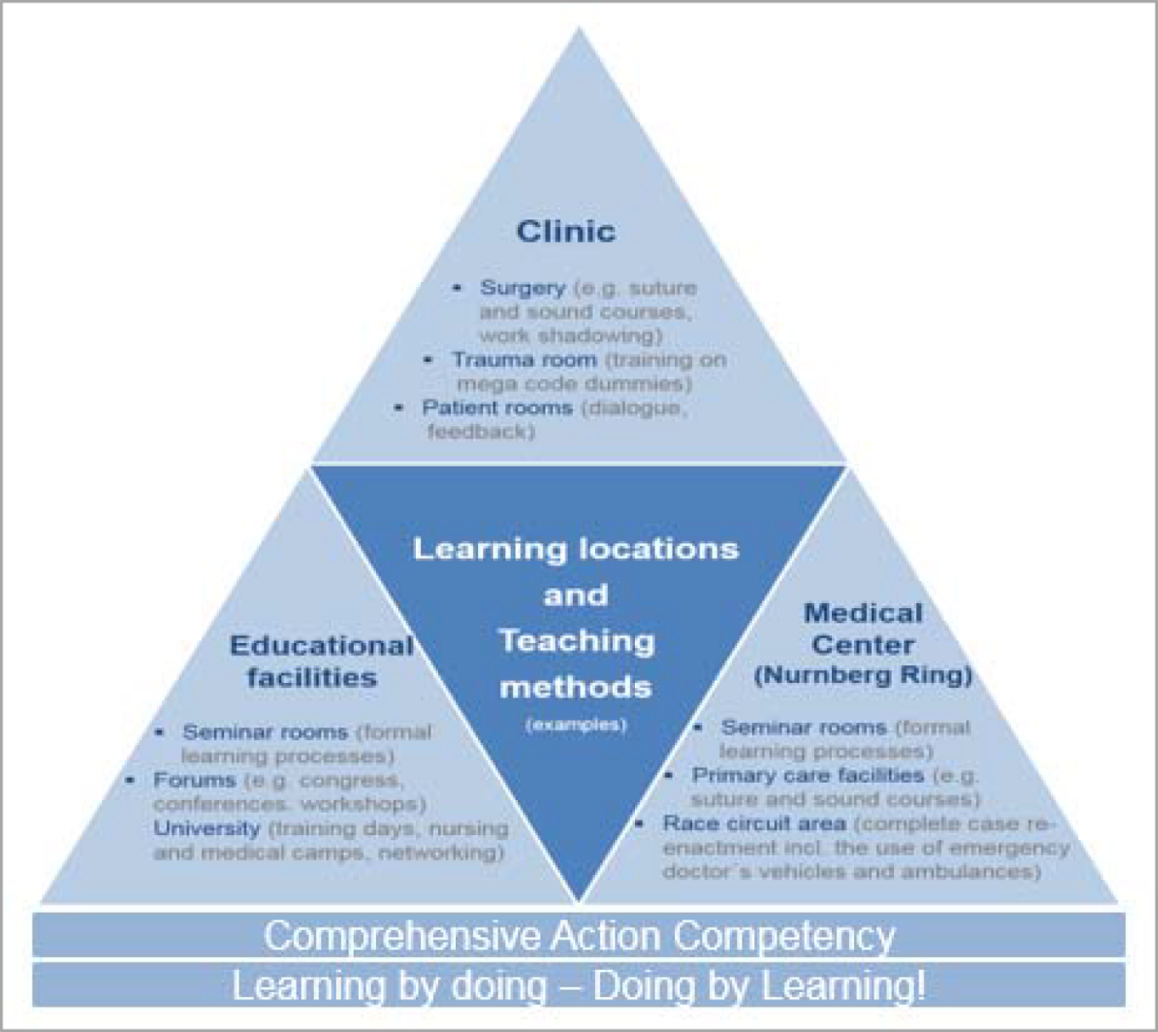
Development of comprehensive action competency – Learning locations and teaching methods

Simulations, planning games, role-play and exercises help to consolidate and deepen theoretical knowledge. On a contextual level, there is a clear link between the selected medium and the everyday professional life of the learners. The medial method of knowledge transfer using, amongst others, e-Learning/blended learning, should not be excluded by the modular system. It has to be stressed that competency at this level is not yet fully developed. Only after overcoming real challenges in practice, where the acting party is intellectually and emotionally challenged, can competency be developed. Practice-relevant scenarios enable not only an exchange among colleagues, but also raise awareness for solutions and perceptions. Individual knowledge is thus expanded through the social dimension of learning in the context of (desired) values, rules, behaviour patterns, emotions and motivations [4]. A comparison between the strategic and content-related goal dimensions of learning, as presented in table 1 (Tab. 1), illustrate this. Whereas figure 6 (Fig. 6) draws attention to structurally learning-friendly conditions.

##### 3.3.3. Transfer of knowledge into practice

The transfer of knowledge into practice represents a first step in competency development taking emotional components in the decision-making process into account [4]. In contrast to knowledge application within re-enacted common scenarios, a doctor‘s individual actions within his field of activity always carry consequences for subsequent actions. In this process, the junior doctor is supported in part by mentoring programmes or by his supervising doctor. It should be noted that doctors providing further training exchange information with the MAW, and are familiar with the requirement for sustained practical use of new knowledge.

##### 3.3.4. Competency development

Other people’s behaviour is regarded as competent when the behaviour they display is judged by a third party to be “effective”. Competency building essentially comprises the internalisation of values. Through processes of reflecting on one’s own role and the role of others, values, norms and rules of individual and social action within the context of the situation are analysed and evaluated. Coaching by colleagues, assessment and feedback discussions, fireside evenings with managers and heads of staff, projects for shaping the future, project work or quality circles in the form of specialised group meetings, are all imperative for successful development. As a basis for action, the reflection result is subordinate to subsequent actions; situational actions are varied if necessary using the options for action identified within reflection.

## 4. Results – Target Achievement and Added Values of the Modular System

The interests of all those involved define effective training processes. To assess whether a training measure has achieved its goal of contributing towards the development of comprehensive medical competency, the modular system bases itself on, amongst other things, the evaluation model on four levels according to Kirkpatrick [16]:

1. Results: which goals and expectations does/do the partner/ the institute/ the participants have?
2. Behaviour: what do success-critical behaviour patterns looks like?
3. Learning: what and how should content be taught in order that participants act accordingly?
4. Satisfaction: which framework conditions are required for the satisfaction of all involved?

In other words, an event is established according to the demands of the didactic triangle: teacher – student – educational content, according to overall objectives. In addition to these fundamental issues of the organisation of competency-enhancing training processes, a methodical triangulation has proved effective in the evaluation process. Here, use is increasingly made of qualitative education controlling, in other words a dialogue-oriented approach within quality assurance. It is geared towards both participants and teachers whereby no feeling of “control” is established. Furthermore, it supports needs orientation in the design of new offers. Depending upon the type and duration of the event, evaluation design can be either formative or summative [17].

**Figure 7:**
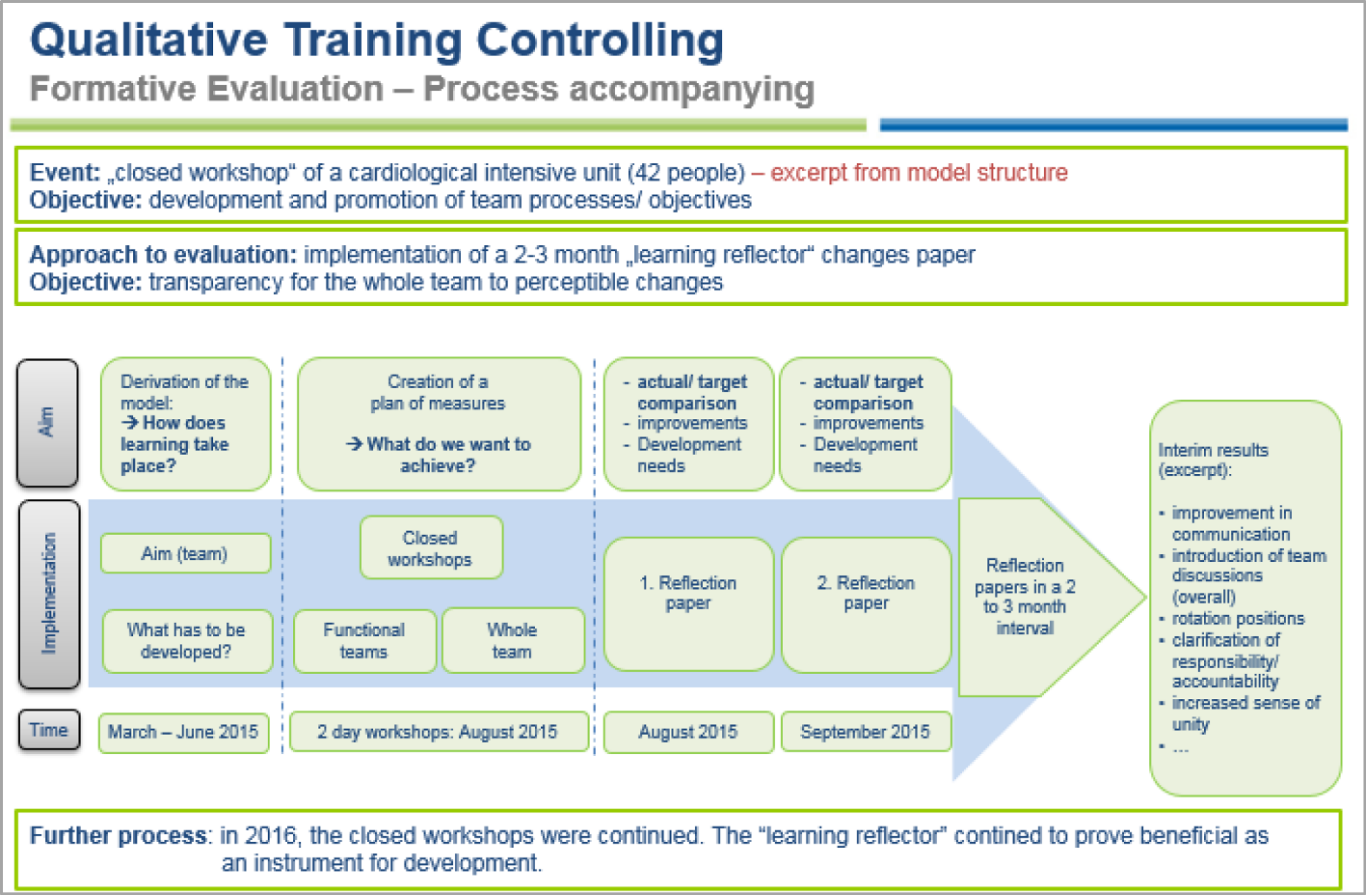
Qualitative training controlling, formative evaluation (excerpt)

The goal of formative evaluation is to continue to support the educational process [17]. The level of learning transfer in particular can then be ensured actively through mentoring programmes, or through instruments for self-assessment (e.g. learning diaries, portfolios, learning reflectors, …) (see figure 7 (Fig. 7)). “Pocket cards” as “lived checklists” for behaviour in everyday working life, e.g. “the Muslim patient – what should I know/be aware of?” or “Culture2Go – management culture at a glance”, were assessed as being useful and urgently recommended for adoption by both participants and instructors in the context of oral evaluation (“learning nuggets” with 6 participants and workshops with 16 participants). At the same time, the curricular structure of the modular system also ensures the associated protection of learning objectives, for example through “learning nugget chains” with an increasing level of performance (e.g. “discussions” through to “conflict talks”). Communicative processes, in particular, were assessed as effective tools for the self-organised further development of individual potential (see also the University of Heidelberg’s curriculum model communication in medicine: http://www.medizinische-fakultaet-hd.uni-heidelberg.de/Medi-KIT.108137.0.html, last accessed 22/7/2016) (see table 3 (Tab. 3)). An experience report on the Central Introductory Seminar (CIS), states that: “In our clinical routine, we will profit routine from lively exchanges with instructors as well as amongst ourselves”; or: “Even the initially seemingly very dry topics such as medical law and labour law were, thanks to friendly instructors […], extremely interactive and interesting”. Furthermore, access via surveys on expectations and experiences have proved effective (“even our requests for special topics were catered for”, excerpt: progress report CIS). Exchange factors between mentor and mentee, or in the form of quality circles, should be noted (“and in the evenings we were able to talk to several experienced senior consultants […] and ask questions”, excerpt: progress report trainees and students in their clinical internship year camp, 2015).

**Figure 8:**
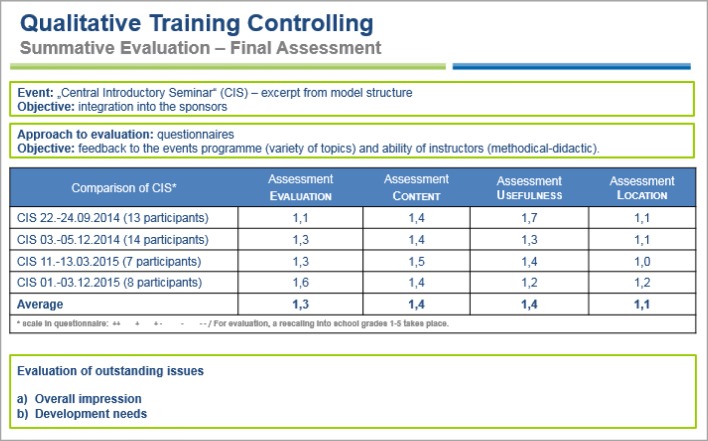
Qualitative education controlling, summative evaluation (extract)

The objective of summative evaluations is a final assessment [17]; they also complement formative evaluations. In this way, each event is assessed through evaluation sheets that are filled out anonymously by participants. As well as on the personal assessment of learning success, the focus here is on feedback on the methods and techniques used by the instructors. This is followed by internal benchmarking. If the results of the “central introductory seminar” are considered, then a development in the areas of “organisation/general conditions”, “content”, “practical benefit” and “seminar venue” can be effectively controlled (see figure 8 (Fig. 8)). Based on feedback that there should in part be stronger practical relevance, the didactic concept could be reappraised with the respective instructor and adjusted through both practical examples and case studies, for example with the thematic block “medical law” (oral evaluation CIS 22.-24.9.2015, 13 participants (cf. figure 8 (Fig. 8)). Closed meetings/team workshops on generic topics such as “leadership and team”, “team building” or “culture workshops for managers” that are organised with ‘complete’ interdisciplinary teams or with function groups, point to the necessity of never forgetting “soft” topics in day-to-day work. In dialogue, profile evaluations with the same teams pointed to an increased sense of togetherness, a pleasant work environment, and the establishment of a positive structure of feedback and debate (see for example formative evaluation figure 7 (Fig. 7)). 

## 5. Discussion – Strengths and Weaknesses

Based on recent discussions about public and private medical schools [see http://www.wissenschaftsrat.de/download/archiv/5100-16.pdf, January 2016], and on the simultaneous revision of further training curricula centred around competency-oriented learning goals (see MBWO), the question is to what extent quality standards can, or even should be operationalised, including in the advanced field of medical competency development. Here the focus is not on the quantity of continuing learning, but rather on its quality with regard to the desired learning goals (knowledge, specific behaviour, increase in patient numbers, …). The strength of the modular system lies in its two-dimensional construction. Development needs can thus be developed individually according to professional position. At this point, the development needs of the model per se are concealed: until now, offers have been generated according to the presumed performance levels of professional positions. At present, a ground-breaking competency model is being devised that carries out a personalised analysis of existing needs by operationalising, assessing and clearly presenting already existing skills. One further didactic strength is that of the instructor/training personnel in their role as “learning guides” [10] and “feedback providers”, who both support and demand the inter-generational transfer of knowledge in practical learning situations. Feedback methods in particular run like a common thread through the events, in the sense of “leaving the protective cocoon of self-confidence” [18] and actively discovering one’s own potential based upon feedback, as well as being shown one’s own fields of learning. The issue up for discussion is the extent to which the instructors/training personnel themselves are trained in didactic and methodological skills – in the modular system this takes place in regular and/or needs-oriented evaluation and coaching discussions with the academy’s management (educational researchers). New events or instructors are always supported by the academy. Further offers aimed specifically at speakers, for example in the form of “train the trainer” events, are being planned. Modular integration of the learning units also allows a prompt and needs-oriented implementation of necessary contents. This is seen as advantageous, in particular with regard to the qualification and development of foreign-trained doctors or career-specific elements. Development needs are visible in the summative evaluation. Perspectively, a random second evaluation of the group of participants after approximately six months is therefore conceivable – for example: “Which contents/incentives of the event have you been able to try out in your working day, what proved to be less useful in practice?” To sum up, the modular system of the Medical Academy Waldbreitbach allows goal-oriented, but nevertheless specific learning – both for the individual as well as for the entire institution. The transparent overview of knowledge and its ‘sponsors’ is both demanded and supported; this contributes towards an increasing dynamism of knowledge and innovation within the organisation.

## 6. Conclusion

It has to be emphasised that competency development should not be regarded as an isolated process. Due to its multidimensional character, competency building is not possible without knowledge (e.g. from guidelines alone), even though knowledge itself does not represent competency. The strength of the modular system is its two-dimensional structure, on the one hand subject-oriented, and on the other geared completely towards the areas of competency. In this way a closer link between the individual positions is strategically achieved, whilst at the same time generating new fields of learning and content. Operationally, a needs-oriented range of short to multi-day learning/training units that can be incorporated effectively into the use of multiple places of learning. With the objective of continuing competency development, the didactic perspective, in other words the use and benefit of various forms, methods and places of learning, is given high priority; this increases individual motivation through real-world needs. Finally, the modular form of individual fields of competency development (from entry level to expert) serves to change the architecture of individual and collective learning thus providing the sustained consolidation of the individual capacity for action.

## Competing interests

The authors declare that they have no competing interests. 

## Figures and Tables

**Table 1:**
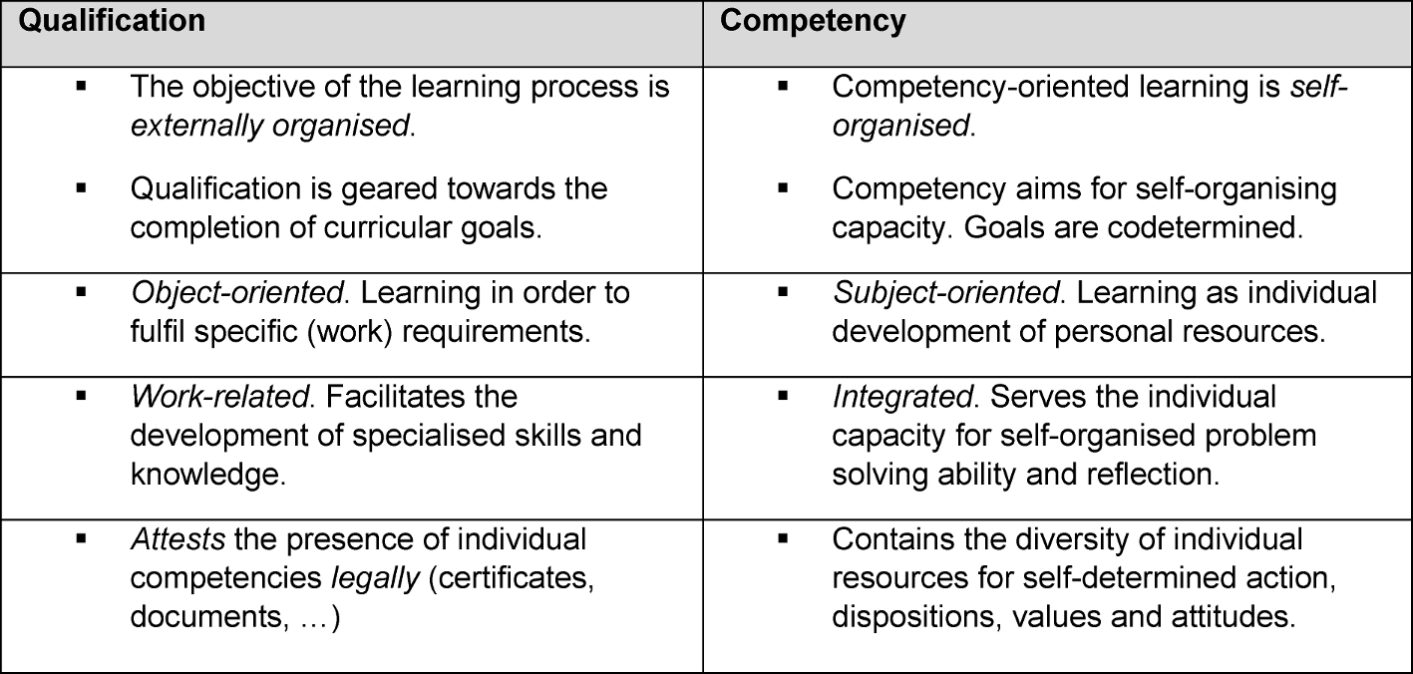
Comparison of qualification and competency [following Erpenbeck/Sauter 2015, [4]]

**Table 2:**
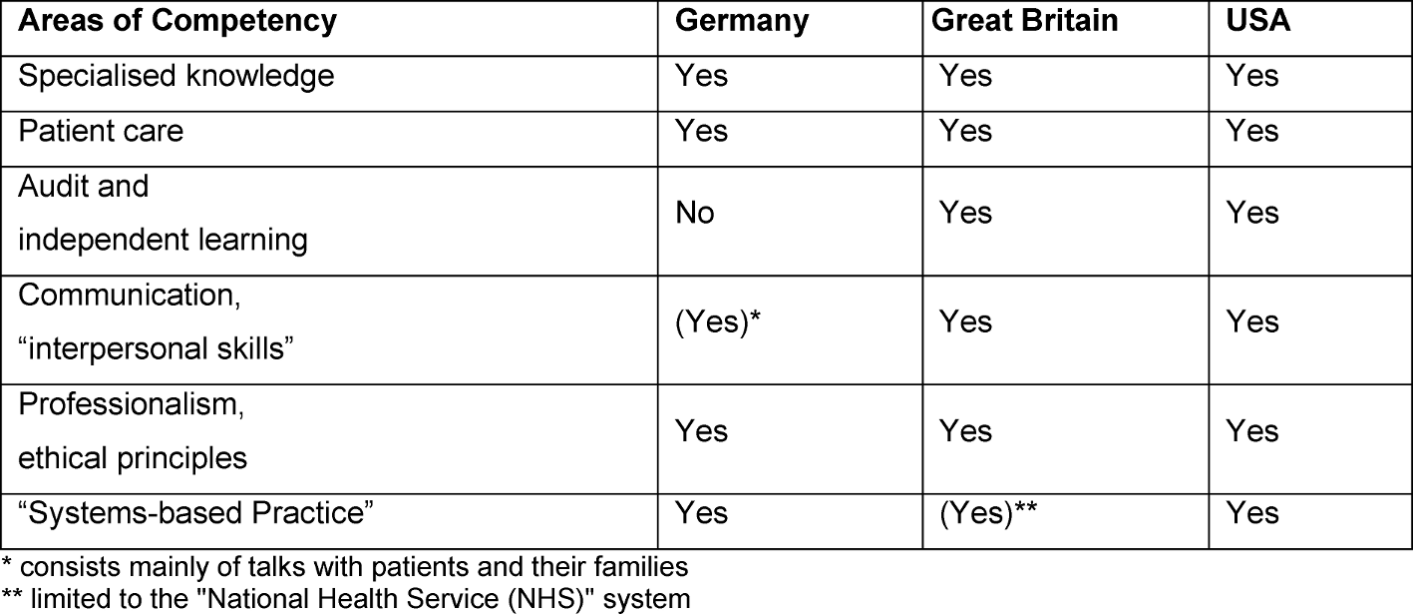
Areas of competency in the further training curricula of various countries.

**Table 3:**
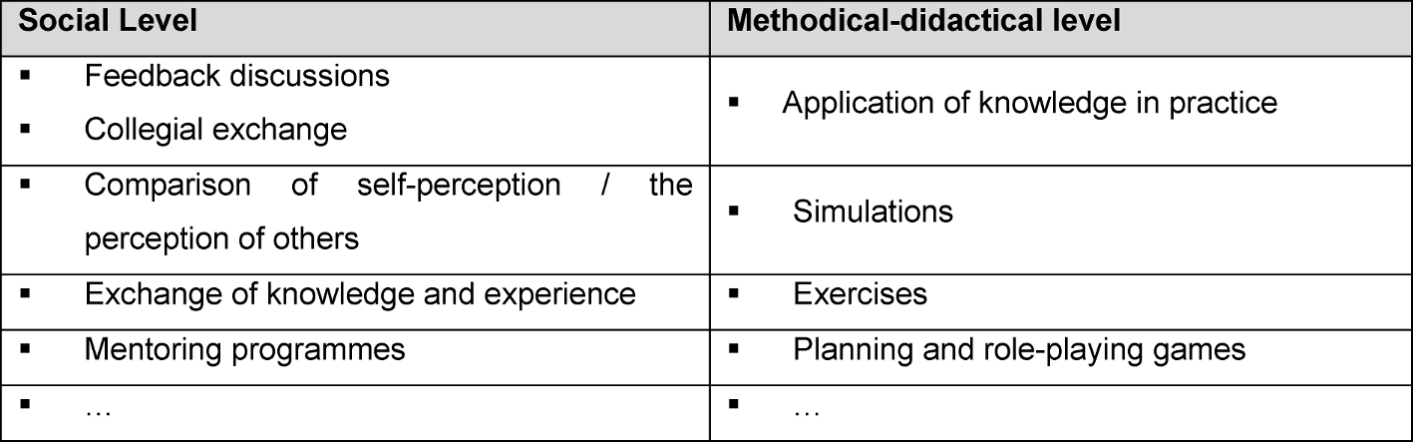
“Tools” for the development of medical competency
